# I Need a Doctor, Call Me a Doctor: Attachment and the Evaluation of General Practitioners before and during the COVID-19 Pandemic

**DOI:** 10.3390/ijerph18157914

**Published:** 2021-07-26

**Authors:** Dritjon Gruda, Konstantinos Kafetsios

**Affiliations:** 1 School of Business, National University of Ireland Maynooth, Maynooth, Ireland; jon.gruda@mu.ie; 2 School of Fine Arts, Aristotle University of Thessalonki, 54124 Thessaloniki, Greece

**Keywords:** adult attachment, social perception, medical professionals, COVID-19, experiment

## Abstract

Attachment is a system of threat regulation, and insecure (anxious and avoidant) attachment orientations are important individual difference antecedents to the cognitive and affective attributions of trait inferences. However, little is known about how threat-related contexts, such as the current COVID-19 pandemic, influence attachment-related socio-cognitive schemas. Using an experimental research design across two independent samples of 330 (pre-onset of COVID-19) and 233 (post-onset of COVID-19) participants, we tested whether attachment orientations influenced general practitioner (GP) ratings and selection differently pre- and post-onset of the COVID-19 pandemic. We found that during the COVID-19 pandemic, when presented with only negative information signals, avoidant individuals attributed positive ratings to GPs, with differing ratings as the number of positive signals increased. Differences between pre- and post-onset of the COVID-19 pandemic were less pronounced with regards to positive signals. We discuss these results in line with signal detection theory (SDT) and provide practical implications in response to our findings.

## 1. Introduction

The COVID-19 pandemic has resulted in an unprecedented worldwide hostile psychological environment [1], while adverse mental health effects, such as anxiety and acute stress disorder [2], and the effects of associated governmental lockdowns and restrictions likely will be felt for months to come. However, in addition to struggling with imposed lockdowns and restrictions, which have changed our lifestyles and perceptions of the world, the COVID-19 pandemic has also changed perceptions of medical professionals.

The perception of positive versus negative social stimuli is important for social behavior [3], especially signals associated with facial emotion expression characteristics [4]. For example, the automatic processing of trust signals in particular is a vital process for human survival [5], as it protects individuals from potential danger [6]. If this automatic process did not exist, there would be a lack of crucial information as to whether someone is a friend or a foe [7]. People are therefore likely to redirect their attention onto the perceived person and subsequently engage in approach or avoidance behavior. The present study focused on positive and negative ratings of GPs, which can influence individuals’ social perceptions consciously or unconsciously, especially in situations of threat such as the COVID-19 pandemic [8].

At a broad conceptual level, as an evolutionary developed behavioral system of fear and uncertainty regulation [9], attachment organization is particularly relevant to the perception and evaluation of positive versus negative social stimuli [3]. Whereas at the distal level, the goal is to increase prospects of survival, proximally, attachment organization is a system of fear and uncertainty regulation. Attachment is activated by exposure to an impending threat and activation of the attachment system motivates searching for affinity and support from important others, and influences individuals’ perception of incoming information [10].

The present study examined the effects of attachment schemas on information processing under the condition of epidemic threat. Attachment-related patterns of threat regulation and social behavior are affected by societal-level crises [11] including the COVID-19 epidemic [12], and we aimed to look at the association of pandemic threat and attachment-related information processing. Information processing during the pandemic is likely different than in non-threatening times [13], as individuals during a time of crisis are likely to rely on available information and individual differences or traits. Albeit generally adaptive for reacting to pathogen stress, the behavioral immune system can sometimes lead to serious biases in social perception [14].

### Attachment and Threat Evaluation

In adulthood, situations of personal or interpersonal distress activate attachment schemas with cognitive and affective consequences [15]. Attachment organization involves anxious and avoidant attachment, two secondary attachment strategies associated with distinct differences in information processing [16]. Adults who score higher on attachment anxiety typically adopt hyperactivating strategies to regulate the anticipated or felt distress. Individuals with avoidant attachment schemas, on the other hand, deactivate the attachment system [17], with consequences for information processing [10]. For example, avoidance has been consistently associated with suppressing and limiting accessibility to attachment-related emotional memories and thoughts about an event, and the reduction of affective hyperactivation as a result of such memories e.g., [15,18]. Yet, avoidant individuals tend to shift between different modes of processing depending on the emotional information and the interpersonal goals [19].

Both anxious and avoidant attachment orientations involve a heightened negativity bias (i.e., greater attention is directed toward threat-related information) and constitute individual differences in processing and exhorting cognitive control of emotional cues from facial appearance [16]. For example, studies looking specifically at detecting emotional cues and anxiety [20] have found that anxiously attached individuals are prone to worse global information processing. Anxious attachment triggers hyperactivation, focusing on “the processing of congruent negative-related cognitions” [21]. Should the attachment behavioral system not be able to regulate the associated threat-related information, the ongoing hyperactivation will inevitably result in weak information processing [20]. Hyperactivation in anxiously attached individuals has been observed in experiments using both positive and negative attachment-related and attachment-unrelated words as well as emotional faces [22].

Avoidant attached individuals also react more strongly to negative pictures than to positive pictures [23]. Due to the negativity bias in avoidant individuals [24], they are prone to favor detecting threat-related information early and are likely to ignore any emotional cues of appraisal [25]. Deactivation increases self-control and reliance in dealing with the received information [21]. Should deactivation be successful, avoidant attached individuals will not be overwhelmed by the perceived emotional cues [18,26].

Based on this heightened negativity bias towards others [25], and physicians in particular [27,28], one would expect insecure individuals to negatively perceive medical professionals. However, in the context of worldwide crises, such as the COVID-19 pandemic [2], we argue that insecure individuals perceive relevant attachment figures, such as physicians, as more secure, to cope with the pandemic threat. Such motivated socio-cognitive mechanisms are known to operate as a response to security regulation. For example, security primes reverse the effects of threat uncertainty on preferences for a strong leader [29]. Such effects are also evident within secondary attachment strategies. Avoidant participants in a dating relationship were less accurate in perceiving positive emotional signals (i.e., happy facial expressions), in comparison to their single counterparts, most likely due to relationship-specific activated higher avoidance [30].

We aimed to test the effects that epidemic threat had on GP perceptions in conditions of either an absence of, or increasing positive signals regarding those GPs. We anticipated that meaningful, secondary-attachment strategies consistent effects would be observed as a function of the absence or number of positive signals. We adopted a two-dimensional approach to measuring followers’ attachment orientations [31] and included an interaction between anxious and avoidant attachment in the same model (see [32]).

## 2. Methodology

Firstly, we collected online data from actual GPs in New York City. Collected data were restricted to GPs, to ensure the data were not tainted, due to participants’ expectations of certain medical professionals. For example, the expectations of psychiatrists and surgeons would likely be quite different.

### 2.1. Pre-Test

Based on 40 unique and randomly selected doctor profiles (https://zocdoc.com, accessed on 13 June 2021), which included images of doctors’ faces, previous patient ratings, the number of comments, and medical school education, we selected two doctors’ profiles to constitute our experimental high and low condition. Both pictures were selected as they were very similar in terms of facial expression and emotional valence, with a neutral background. Both conditions were pre-tested to ensure participants correctly identified both vignettes as either highly likable or less likable. Subsequently, 103 U.S. participants on Amazon’s Mechanical Turk (MTurk) platform were shown, in a random order, one of the two conditions (0 = low likability, 1 = high likability) and were provided with comparable compensation for their time and effort. Results showed that the mean of the highly likable GP was higher than the less likable GP (M = 3.15, SD = 0.09 vs M = 3.51, SD = 0.07, *t* = −3.22, *p* = 0.015). These results suggest that the manipulations had their intended effects.

In total, the final sample included 553 participants from the United States (pre-COVID-19 cohort: 330 participants; COVID-19 cohort: 223 participants), who were recruited via Amazon’s Mechanical Turk to take part in our experiment. All participants passed several attention check questions [33].

### 2.2. Pre-Onset of COVID-19 Experiment and Questionnaire

Participants were instructed to imagine they had moved to a new town and were browsing online to find a good GP for themselves and their families, and to provide an overall rating of the displayed GP profile. Each presented GP profile was composed of four components, namely, an image of doctors’ faces, previous patient ratings, the number of previous patients’ comments, and the rank of the attended medical school. Each element was either displayed as a high or low condition.

Since four elements (high/low) were shown randomly, participants were randomly shown zero to four “positive signals”. We define “positive signals” to constitute the sum of high conditions displayed to participants. Therefore, a minimum sum of zero positive signals did not only constitute a lack of positive signals, but also the display of four negative signals. Subsequent to this information, participants provided their ratings and completed several measures of individual differences, including attachment orientations, Big Five personality dimensions, and demographics. Finally, participants were debriefed and thanked for their participation.

#### 2.2.1 Measures

##### Overall Doctor Rating

Participants were asked to provide a rating score of the presented GP, based on the random information presented during the experimental task. The single item was phrased as “Given all you know now, how would you rate this doctor?” and was measured based on a single-item scale from 0–5.

##### Attachment Orientations

Individuals’ attachment was assessed using the adapted Experience in Close Relationships scale (ECR) developed by Richards and Schat [34], based on the work of Brennan, et al. [35]. The ECR consists of 36 items on two subscales measuring anxious attachment (α = 0.96) and avoidant attachment (α = 0.96) on a 7-point scale ranging from 1 (“strongly disagree”) to 7 (“strongly agree”).

##### Personality

We controlled for personality traits as defined by the International Personality Item Pool Five-Factor Model (mini IPIP) scale [36]. The five factors, openness to experience (α = 0.83), conscientiousness (α = 0.79), extraversion (α = 0.90), agreeableness (α = 0.86), and neuroticism (α = 0.81) were assessed on a 5-point scale ranging from 1 (“very inaccurate”) to 5 (“very accurate”) for all dimensions.

##### Demographics

Assessed demographic data included age and gender. We expected that participants’ gender might play a role since selected pictures in the conducted experiment displayed male GPs.

### 2.3. Post-Onset of COVID-19 Experiment and Questionnaire

Instructions for participants in the post-onset of COVID-19 cohort were identical to the pre-onset of COVID-19 cohort, with one exception. At the beginning of the experiment, participants in the COVID-19 cohort were first asked to think back to the onset of COVID-19 in their local region/city and to complete a measure that indicated how COVID-19 had affected them [37]. By including this section before the main task and individual differences measures, we ensured that all participants thought about their first experiences of the COVID-19 pandemic irrespective of the actual date of their first exposure to the pandemic and its social and economic repercussions. We also included this variable as an additional control in robustness checks of the final model.

### 2.4. Measures

Identical measures as presented in the pre-COVID-19 sample.

#### Fear of Coronavirus-19

Participants in the COVID-19 cohort completed the fear of coronavirus-19 scale [37]. This scale is composed of 7 items (α = 0.67) and includes statements such as “It makes me uncomfortable to think about coronavirus-19” and “My hands become clammy when I think about coronavirus-19”. Participants recorded their responses on a 5-point scale ranging from 1 (“strongly disagree”) to 5 (“strongly agree”).

## 3. Results

Correlations and reliability alphas are reported in Table 1 and the main results are reported in Table 2 below.

Using ordinary least squares (OLS) regressions with a heteroscedastic-robust estimate of the variance, we regressed the dependent variable (i.e., the overall rating attributed to the described medical professional) on the sum of displayed positive, anxious, and avoidant attachment. Since we were mostly interested in the differences between participants in the pre- vs. post-COVID-19 onset group, we ran a four-way interaction model. Doing so allowed us to compare participants in both groups within the same model, avoid losing variance, and helped to decrease statistical noise.

As stipulated, compared to zero positive signals, anxious attachment (e.g., two positive signals: *b* = −2.38, *t* = −2.78, *p* = 0.006) and avoidant attachment (e.g., two positive signals: *b* = −1.48, *t* = −2.21, *p* = 0.027) significantly predicted participants’ overall rating of GPs pre-and-post the onset of COVID-19 (Table 3).

**Figure 1:**
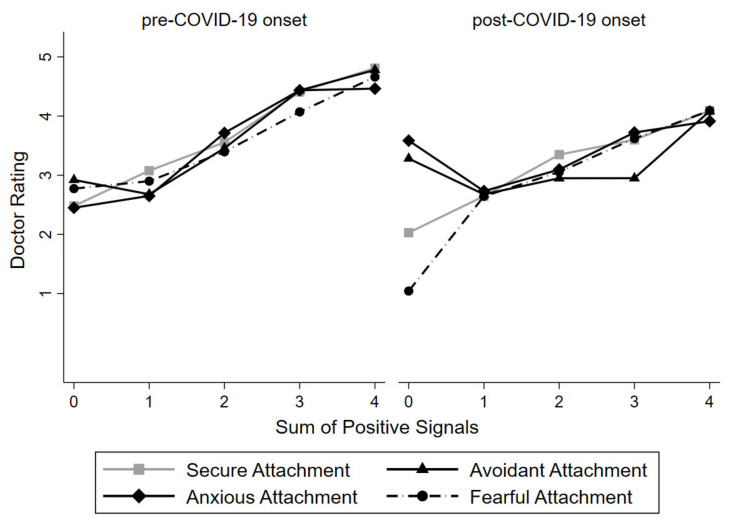
Attachment orientations and doctor rating by signal (pre-and-post the onset of COVID-19)**.** Note: positive signals = sum of displayed high conditions (i.e., picture, ranking of medical school, number of previous patient comments, previous patient rating); secure attachment = low anxious and avoidant; anxious attachment = high anxious/low avoidant; avoidant attachment = low anxious/high avoidant; fearful attachment = high anxious/high avoidant); *n* = 330 (pre-onset of COVID-19), 223 (post-onset of COVID-19).

Given the complexity of this interaction, and to understand this interaction better, we graphed our main interaction results (+/− 1 SD) in Figure 1, which visualizes results pre-and-post the onset of COVID−19 and by signal condition, respectively.

Based on these results, it seems the greatest differences in the influence of attachment orientations pre- and post-onset of COVID-19 were found in the case of zero positive signals. A simple slopes test showed that compared to the pre-COVID-19 condition, highly anxious individuals in the COVID-19 cohort rated doctors highly despite the lack of any positive signals (zero positive signals: simple slope = 1.66, *t* = 2.70, *p* = 0.007). Results were marginally significant for highly avoidant individuals in the COVID-19 cohort (zero positive signals: simple slope = −0.98, *t* = −1.83, *p* = 0.068). However, in the case of three positive signals, when compared to the pre-COVID-19 condition, highly avoidant individuals in the COVID-19 cohort rated doctors lower despite the evidence of several positive signals (three positive signals: simple slope = −1.04, *t* = −2.94, *p* = 0.003).

Finally, as a robustness check, we also ran two separate regression models, one for each (pre- and post-onset of COVID-19) cohort. In a separate step, we also controlled for fear of coronavirus in the COVID-19 cohort and Big Five personality traits. In both instances, results remained largely unchanged. Additional information is available by the authors upon request.

## 4. Discussion

The COVID-19 pandemic has created a unique hostile psychological environment with adverse mental health effects to likely follow for years to come. However, the COVID-19 pandemic is different from previous crises in that although it seems to have affected entire populations [38], individual differences such as personality traits have also influenced individuals’ affects and perceptions of others [2].

During this time of crisis, negative signals, or the absence of positive signals (i.e., less well liked facial features, less highly ranked medical education, lower number of patient comments, and lower previous patient ratings) provide a different basis of trait inferences for individuals with a dominant anxious and avoidant attachment orientation regarding the rating and selection of medical professionals.

Based on the interface between attachment theory and signal detection theory, we argue that our results are likely due to the introduction of additional information or signal conditions. Although the sample of participants in each cohort was not the same, and therefore causal statements are difficult, as participants were shown different pieces of information (high/low condition) in a random order, we could account for the differences that additional information has on the relationship between attachment orientations and overall GP ratings between the two cohorts.

In the post-COVID-19 onset cohort, in the complete absence of any positive signals, avoidant individuals provided higher ratings of GPs; once avoidant individuals were presented with any combination of positive and negative signals, they seemed to require several more positive signals to provide more favorable GP ratings than anxious individuals. These findings are in contrast to results in the pre-COVID-19 cohort, in which ratings increased linearly with a higher number of positive signals regardless of attachment orientation. Therefore, the post-COVID-19 pandemic findings cannot be explained with avoidant individuals’ tendency to shy away from the threat and negative emotional information and a selective reduction of pleasantness ratings for positive social information [39]. Rather, we argue that in times of crisis, in line with social motivated cognition theory [24], avoidance may involve adaptive processes including social information processes. Put differently, it seems that in the presence of the COVID-19 pandemic threat, and the associated negative cognitive and emotional load [40,41], avoidant individuals seem to fail to ignore attachment-relevant information and provide higher ratings of presented GPs in the complete absence of positive information. As stipulated by Sakman and Sümer [41], avoidant individuals seem to also be preoccupied with attachment-related thoughts and emotions, similarly to anxious individuals. However, in comparison to anxious individuals, avoidant individuals have learned to suppress this information, a process that requires cognitive capacity and resources. Additional cognitive load, for example, the threat of a pandemic, seems to lead to failure of the aforementioned suppression process.

On the other hand, during the COVID-19 health crisis, anxious individuals also provided more positive GP ratings in the presence of less favorable GP information. It seems that anxious persons, those who depend on others more greatly to provide support and to be a safe haven, came to more positive ratings during the pandemic when it came to choosing a new, trusting, and competent doctor for themselves and their families. This is likely because the increased uncertainty regarding the COVID-19 pandemic and its repercussions forced them to engage in hyperactivating behavior and effectively seek out proximity and support in others. This is likely the case because anxious attachment is associated with an impaired capacity to regulate their experienced uncertainty and processing [42].

We understand these results in line with a motivated social cognitive account of avoidant attachment strategies [29] applied to emotion perception. Therefore, in the experiment, both anxious and avoidant attached participants in the COVID-19 cohort sought proximity and a safe haven by embracing and highly rating the presented GPs when presented with a complete lack of positive signal information. However, once presented with at least one positive signal, individuals with different attachment orientations differed in their responses. In addition, and following recent work on attachment and sentinel behavior [43], it could be that anxious individuals are more likely to embrace doctors even in the absence of positive signals to protect themselves and their families. On the other hand, avoidant attached individuals might be more likely to protect themselves first, and in the absence of positive signals are willing to accept any doctor due to the experienced additional cognitive load requiring them to decide on a medical practitioner. However, once avoidant individuals are presented with at least one positive signal, they require a higher number of positive pieces of information about the respective doctor to provide a high overall rating.

Medical professionals should be aware that patients’ perceptions of them might not be related to their medical or analytical skillset, but rather can be based on individuals’ relational traits, embedded in their (in)dependency on others, and context. If a medical professional is perceived as less likable due to information readily available online, potential patients might not consider this doctor, although the doctor might be perfectly feasible to examine and treat the patient. Understanding that potential patient perceptions are influenced by underlying relational personality factors, such as attachment orientations, is vital and can help to better explain the perception and attribution of negative/positive characteristics towards medical professionals, especially during trying times such as the current COVID-19 pandemic.

## 5. Conclusions

In this manuscript, we examined attachment orientations as possible antecedents in the cognitive and affective attribution of characteristics to GPs before and after the onset of COVID-19. While both anxious and avoidant attached participants in the COVID-19 cohort rated GPs highly even when participants were shown no positive information regarding the respective GP, these results did not replicate in the pre-COVID-19 cohort. We discussed possible implications per signal detection theory. Finally, this study emphasized the importance of attachment orientations, relational traits, and context in explaining the perception and information processing of characteristics related to medical professionals.

## Figures and Tables

**Table 1:** Correlations between main variables (pre-COVID-19 onset).

		M	SD	(1)	(2)	(3)	(4)	(5)	(6)	(7)	(8)	(9)	(10)
(1)	Doctor Rating	3.57	1.05	-									
(2)	Positive signal	1.96	1.01	0.56 ***	-								
(3)	Anxious Attachment	2.82	1.31	−0.01	0.05	(0.96)							
(4)	Avoidant Attachment	3.66	1.37	−0.05	0.00	0.43 ***	(0.96)						
(5)	Openness to Experience	3.93	0.91	0.00	0.04	−0.16 ***	−0.15 ***	(0.83)					
(6)	Conscientiousness	3.90	0.86	−0.06	−0.05	−0.39 ***	−0.22 ***	0.13 *	(0.79)				
(7)	Extraversion	2.55	1.15	−0.00	−0.03	−0.27 ***	−0.52 ***	0.31 ***	0.17 ***	(0.90)			
(8)	Agreeableness	3.84	0.90	−0.04	−0.00	−0.17 ***	−0.56 ***	0.27 ***	0.19 ***	0.33 ***	(0.86)		
(9)	Neuroticism	2.25	0.96	0.00	0.07	0.59 ***	0.38 ***	−0.15 **	−0.46 ***	−0.29 ***	−0.15 **	(0.81)	
(10)	Age	4.68	0.77	−0.01	0.02	−0.21 ***	−0.04	−0.01	0.12 *	0.00	0.06	−0.17 **	-
(11)	Gender	0.55	0.50	−0.03	−0.07	−0.01	0.07	0.08	0.04	−0.00	−0.19 ***	−0.21 ***	−0.09

Note: age: 3 = 18–25, 4 = 26–34, 5 = 35–54, 6 = 55–64, 7 = 65 or over; gender: male = 1, female = 0; Cronbach reliability alphas in parentheses where appropriate; *** *p* < 0.001, ** *p* < 0.01, * *p* < 0.05, *n* = 330.

**Table 2:** Correlations between main variables (post-COVID-19 onset).

		M	SD	(1)	(2)	(3)	(4)	(5)	(6)	(7)	(8)	(9)	(10)	(11)
(1)	Doctor Rating	3.58	1.06	-										
(2)	Positive signals	2.0	0.99	0.49 ***										
(3)	Anxious Attachment	2.70	1.27	0.03	0.02	(0.96)								
(4)	Avoidant Attachment	3.57	1.37	−0.10	−0.03	0.39 ***	(0.97)							
(5)	COVID Fear	2.74	1.09	0.01	0.04	0.18 **	0.01	(0.67)						
(6)	Openness to Experience	4.02	0.89	0.08	0.04	−0.13 *	−0.25 ***	0.02	(0.80)					
(7)	Conscientiousness	4.10	0.77	0.03	0.10	−0.44 ***	−0.25 ***	−0.08	0.06	(0.78)				
(8)	Extraversion	2.64	1.14	0.04	−0.05	−0.24 ***	−0.59 ***	0.05	0.33 ***	0.19 **	(0.90)			
(9)	Agreeableness	3.97	0.82	0.13	0.06	−0.20 **	−0.62 ***	0.03	0.30 ***	0.18 **	0.35 ***	(0.84)		
(10)	Neuroticism	2.20	0.93	−0.12	−0.06	0.64 ***	0.39 ***	0.22 ***	−0.30 ***	−0.37 ***	−0.34 ***	−0.21 **	(0.82)	
(11)	Age	4.91	0.87	−0.07	−0.03	−0.25 ***	−0.09	−0.13 *	−0.10	0.05	0.06	0.06	−0.29 ***	-
(12)	Gender	0.50	0.50	0.23 ***	0.09	0.01	0.10	−0.08	0.18 **	−0.01	0.04	−0.26 ***	−0.17 *	−0.11

Note: age: 3 = 18–25, 4 = 26–34, 5 = 35–54, 6 = 55–64, 7 = 65 or over; gender: male = 1, female = 0; Cronbach reliability alphas in parentheses where appropriate; *** *p* < 0.001, ** *p* < 0.01, * *p* < 0.05, *n* = 223.

**Table 3:** Regression interaction between attachment orientations and positive signals pre-and-post the onset of COVID-19. The addition of Big Five personality traits did not change results and were left out due to space limitations. Full information is available from the authors.

	Coef.	*t*-Value	Low 95% CI	High 95% CI
Corona (pre/post)	−3.90 *	−2.28	−7.25	−0.54
Signal (0)	*(base level)*			
Signal (1)	1.87 ^†^	1.88	−0.09	3.8
Signal (2)	1.37 ^†^	1.79	−0.13	2.86
Signal (3)	2.11 **	2.82	0.64	3.58
Signal (4)	3.06 ***	5.59	1.98	4.13
Corona X Signal (0)	*(base level)*			
Corona X Signal (1)	2.85	1.41	−1.13	6.83
Corona X Signal (2)	5.01 *	2.55	1.16	8.86
Corona X Signal (3)	4.65 *	2.50	1.00	8.29
Corona X Signal (4)	3.66 *	2.04	0.13	7.19
Anxious Attachment	0.03	0.24	−0.19	0.238
Corona X Anxious Attachment	2.01 **	2.71	0.55	3.47
Corona X Signal (0) X Anxious Attachment	*(base level)*			
Corona X Signal (1) X Anxious Attachment	−1.57 ^†^	−1.86	−3.22	0.09
Corona X Signal (2) X Anxious Attachment	−2.38 **	−2.78	−4.07	−0.70
Corona X Signal (3) X Anxious Attachment	−2.29 **	−2.91	−3.83	−0.75
Corona X Signal (4) X Anxious Attachment	−1.95 *	−2.54	−3.46	−0.44
Avoidant Attachment	0.19	1.41	−0.07	0.44
Corona X Avoidant Attachment	1.22 *	1.97	0.01	2.44
Corona X Signal (0) X Avoidant Attachment	*(base level)*			
Corona X Signal (1) X Avoidant Attachment	−0.90	−1.32	−2.25	0.45
Corona X Signal (2) X Avoidant Attachment	−1.48 *	−2.21	−2.79	−0.17
Corona X Signal (3) X Avoidant Attachment	−1.71 *	−2.59	−3.00	−0.41
Corona X Signal (4) X Avoidant Attachment	−1.21 ^†^	−1.92	−2.45	0.03
Anxious X Avoidant Attachment	−0.017	−0.56	−0.07	0.04
Corona X Anxious X Avoidant Attachment	−0.59 *	−2.32	−1.09	−0.09
Corona X Signal (0) X Anxious X Avoidant Attachment	*(base level)*			
Corona X Signal (1) X Anxious X Avoidant Attachment	0.48 ^†^	1.76	−0.06	1.01
Corona X Signal (2) X Anxious X Avoidant Attachment	0.68 *	2.47	0.14	1.21
Corona X Signal (3) X Anxious X Avoidant Attachment	0.73 **	2.77	0.213	1.25
Corona X Signal (4) X Anxious X Avoidant Attachment	0.59 *	2.29	0.08	1.09
Constant	1.90 ***	4.14	1.00	2.80
R^2^	0.33 ***			

Note: CI = Confidence Interval; sum of positive signals is categorical; model includes age and gender as additional controls; *** *p* < 0.001, ** *p* < 0.01, * *p* < 0.05, ^†^ *p* < 0.10; *n* = 553.

## Data Availability

The examined dataset is available from the authors upon reasonable request.
